# Dopamine modulates hemocyte phagocytosis via a D1-like receptor in the rice stem borer, *Chilo suppressalis*

**DOI:** 10.1038/srep12247

**Published:** 2015-07-16

**Authors:** Shun-Fan Wu, Gang Xu, David Stanley, Jia Huang, Gong-Yin Ye

**Affiliations:** 1 State Key Laboratory of Rice Biology & Key Laboratory of Agricultural Entomology of Ministry of Agriculture, Institute of Insect Sciences, Zhejiang University, Hangzhou 310058, China; 2 College of Plant Protection, Nanjing Agricultural University, Nanjing 210095, China; State & Local Joint Engineering Research Center of Green Pesticide Invention and Application; 3 USDA – Agricultural Research Service, BCIRL, 1503 S. Providence Road, Columbia MO 65203 USA

## Abstract

Dopamine (DA) is a signal moiety bridging the nervous and immune systems. DA dysregulation is linked to serious human diseases, including addiction, schizophrenia, and Parkinson’s disease. However, DA actions in the immune system remain incompletely understood. In this study, we found that DA modulates insect hemocyte phagocytosis using hemocytes prepared from the rice stem borer (RSB), *Chilo suppressalis*. We investigated whether insect hemocytes are capable of *de novo* DA production. Here we show that exposing hemocytes to lipopolysaccharide (LPS) led to induction of DA-generating enzymes. Exogenous DA induced rapid phosphorylation of extracellular signal-regulated kinase (ERK) in naïve hemocytes. Activation of ERK was inhibited by preincubating with a DOP1 receptor antagonist. Thus, DA signaling via the DOP1 receptor may contribute to early hemocyte activation. DA synthesized and released from hemocytes may act in an autocrine mechanism to stimulate or maintain phagocytic activity. Consistent with this hypothesis, we found that inhibition of DA synthesis with α-methyl-DL-tyrosine methyl ester hydrochloride or blockage of DOP1 receptor with antagonist SCH23390 impaired hemocyte phagocytosis. Topical DA application also significantly decreased RSB mortality following challenge with the insect pathogenic fungus, *Beauveria bassiana*. We infer that a DA-dependent signaling system operates in hemocytes to mediate phagocytotic functions.

The central and peripheral nervous systems modulate immune function by releasing soluble factors such as neurotransmitters and neuropeptides^1^,^2^,^3^. The classical catecholamine neurotransmitter, dopamine (DA), is particularly interesting because in addition to regulating movement^4^, decision-making^5^, drug addiction^6^, pain perception^7^ and endocrine functions^8^, it also modulates immune functions^9^,^10^. For example, DA is synthesized by several immune effector cells and its receptors are present in these cells^11^,^12^. Incubating human lymphocytes with DA inhibited Concanavalin A-stimulated proliferation and synthesis of IFNγ^13^. DA operates in human immunodeficiency virus (HIV) and simian immunodeficiency virus (SIV)-induced brain pathology^14^,^15^, and in lymphoproliferative response^16^. Gaskill *et al.*^17^ reported that DA activated macrophages, which express DA receptors, by increasing ERK 1 phosphorylation. They also showed that DA increased HIV replication in human macrophages via activation of the DA receptor DR2^17^. Several studies have demonstrated that T cells and dendritic cells were functionally responsive to DA, suggesting it modulates immunity^12^,^18^. Stimulation of the DA receptor D5 on dendritic cells potentiates Th17-mediated immunity^19^.

Once released from presynaptic terminals, DA activates D1 and D2 classes of DA receptors in the target cells^20^,^21^. In vertebrates, the D1 class includes the D1 and D5 subtypes, which on activation increase intracellular cAMP. The D2 receptors, including D2, D3 and D4 subtypes, inhibit generation of intracellular cAMP^20^,^21^. Invertebrates express three distinct classes of DA receptors. One is the “DOP1” group, most closely related to the vertebrate D1-like receptors; they activate adenylate cyclase to increase intracellular cAMP^22^,^23^,^24^. Another is the invertebrate-type DA receptors, which increase intracellular cAMP levels and couple with intracellular Ca^2+^ response^25^,^26^,^27^. The third group, the invertebrate D2-like receptors, share most homology with vertebrate D2 class receptors, which decrease in intracellular cAMP levels^28^,^29^.

Phagocytosis is one of the oldest cellular processes, serving as a feeding mechanism, a development process and also as a key defense reaction in innate immunity of all multicellular organisms^30^. Macrophages and neutrophils are the ‘professional’ mammalian phagocytes that internalize senescent and apoptotic cells, as well as invading pathogens^30^. Dendritic cells present processed antigens to lymphocytes, thus linking innate and adaptive immunity^31^,^32^. In insects, the innate immune system is divided into humoral and cellular defense responses. Cellular immune response refer to hemocyte-mediated response such as phagocytosis and encapsulation^33^. Phagocytosis is an important innate immune response against pathogens and parasites^34^. In the lepidopteran insects, the ‘professional’ phagocytes are granulocytes and plasmatocytes^35^. A point of interest is the potential cross-talk between the immune and nervous system may play a role in regulating phagocytosis-like response in insects during infection^33^. However, the specific actions of neurotransmitters in insect phagocytosis are less well characterized compared with vertebrate counterparts.

The major insect cellular immune reactions to infection and invasion are phagocytosis, nodulation and encapsulation, all mediated by a substantial range of compounds, some of which exert similar actions in vertebrate and invertebrate immunity. The similarity provoked us to pose the hypothesis that DA is one the signal moieties responsible for mediating phagocytosis by insect hemocytes. Here we present the outcomes of experiments with the rice stem borer (RSB), *Chilo suppressalis*, designed to test our hypothesis.

## Results

### Bacterial LPS challenge stimulates hemocyte DA synthesis

**Figure 1:**
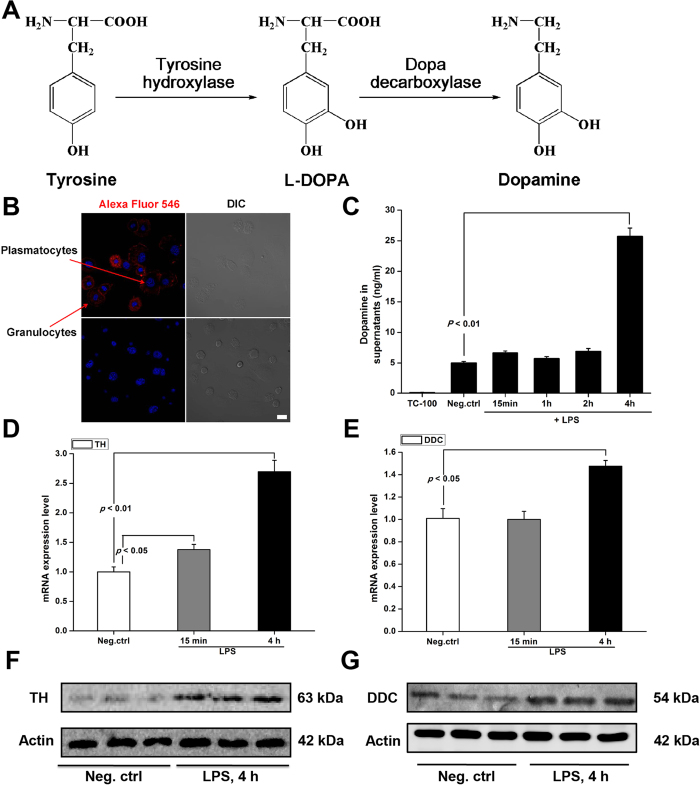
Release of dopamine from phagocytes and the presence of dopamine-producing enzymes in hemocytes. Activated hemocytes are capable of dopamine synthesis. (**A**) Schematic representation of dopamine biosynthetic pathway. (**B**) Dopamine was visualized by confocal microscopy in hemocytes by labeling with dopamine antiserum (Alexa Fluor 546; red) (upper left). Examples of a plasmatocyte and granulocyte are indicated with red arrows. Dopamine antisera was pre-absorbed with DA as the negative control (lower left). Nuclei were counterstained with DAPI (blue). Scale bar represents 10 μm. Data are representative of 3 independent experiments. (**C**) Dopamine concentrations in hemocytes culture supernatants were determined by HPLC-MS/MS. Negative control (Neg. ctrl) served as cells incubated without LPS for 4 h. (**D**,**E**) After isolation and stimulation of hemocytes with 100 ng/ml of LPS *in vitro*, mRNA from hemocytes was isolated and subjected to real-time PCR analysis for tyrosine hydroxylase (TH) (**D**) and dopa decarboxylase (DDC) (**E**). (**F**,**G**) After isolation and stimulation with 100 ng/ml of LPS *in vitro*, protein from hemocytes were subjected to analysis by western blotting for the TH (**F**) and DDC (**G**). Real-time PCR data are presented as means ± s.e.m.; *n* ≥ 6 per bar. Western blots were repeated at least three separate times. Representative blots are shown. Neg. ctrl: negative control.

Figure 1A outlines insect DA biosynthesis, in which the tyrosine hydroxylase step is rate-limiting^35^. Figure 1B shows DA granules labeled with anti-DA antibody in the cytosol of hemocytes (untreated with LPS). To determine whether hemocytes generate and release DA, cells were incubated with bacterial LPS and amounts of DA in cell supernatants were determined by HPLC-MS. Hemocytic DA amounts increased significantly from about 5 ng/ml to over 25 ng/ml in hemocyte supernatants 4 h after exposure to LPS (Fig. 1C). On the basis of these findings, we evaluated the expression of genes encoding tyrosine hydroxylase and dopa decarboxylase by qPCR. Hemocytes were incubated for 15 min and 4 h with 100 ng/ml LPS. To confirm the hemocytes recognized the LPS challenge, we used qPCR to determine expression levels of the genes encoding the anti-fungal peptide defensin and the pattern recognition receptor PGRP-S2 (Figure S1A and B); expression of both genes was substantially induced by LPS. Figure 1D,E show that expression of tyrosine hydroxylase increased by about 2.5-fold and expression of dopa decarboxylase increased by about 1.5-fold at 4 h post LPS challenge. We confirmed our qPCR results by Western blotting; at 4 h post LPS challenge the expression of both proteins was markedly increased (Fig. 1F,G).

### Hemocytes express CsDOP1

**Figure 2:**
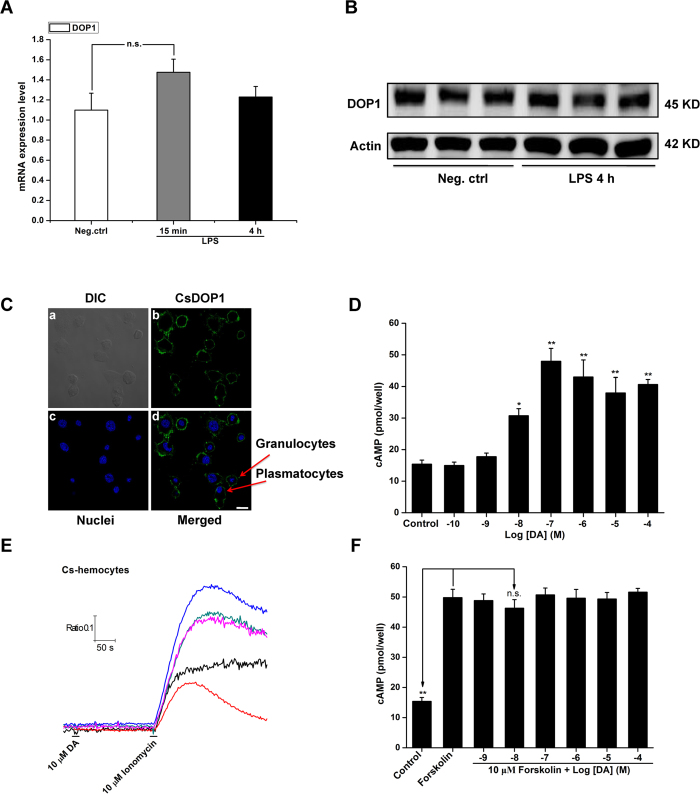
Dopamine receptor subtypes in hemocytes. After isolation and stimulation of hemocytes with 100 ng of LPS *in vitro*, mRNA and protein from hemocytes were subjected to analysis by real-time PCR (**A**) and western blotting (**B**) for the CsDOP1. (**C**) Immuno-localization of CsDOP1 on hemocytes. (**D**) Effects of various concentrations of DA on intracellular cAMP levels in hemocytes. (**E**) Effects of 10 μM DA and 10 μM ionomycin on [Ca^2+^]_i_ in hemocytes. DA was applied for a duration of 3–5 s at the times indicated by black bars. (**F**) Effects of various concentrations of DA on intracellular cAMP levels in hemocytes. Data represent the means ± s.e.m. of at least three independent experiments, each performed in triplicate. The statistical analysis is based on a one-way ANOVA followed by Tukey’s multiple comparison test; ***P ≤ 0.001, **P ≤ 0.01, *P ≤ 0.05.

We tested DA receptor gene expression in hemocytes. Although CsDOP2 and CsDOP3 was also detected by qPCR, the Ct values were larger (Table S2). We analyzed control hemocyte preparations immediately following isolation; and experimental preparations after 4 h exposure to 100 ng/ml LPS. As shown in Fig. 2A,B, control and experimental hemocyte preparations express the CsDOP1 receptor, and levels of the transcript and protein remained constant following LPS-activation. We confirmed the presence of CsDOP1 using immunofluorescence to directly visualize CsDOP1 within hemocytes (Fig. 2C). The figure also indicates that CsDOP1 was expressed on the plasmatocyte and granulocyte membranes. CsDOP1 receptors couple with a G_s_ protein and increase intracellular cAMP concentrations ([cAMP]_i_) in a DA dose-dependent manner, from about 15 pmol/well in controls to about 50 pmol/well after DA treatment at 10^−7^ M and higher concentrations (Fig. 2D).

We ruled out the possibility that the functional of CsDOP2 (increase intracellular Ca^2+^) or a CsDOP3 (decrease cAMP]_i_) operates in RSB hemocytes^28^. We stimulated *in vitro* hemocyte preparations with DA and monitored intracellular Ca^2+^ and, separately, [cAMP]_i_ concentrations. Figure 2E shows that DA treatments (10 μM) did not induce a Ca^2+^ response; Fig. 2F shows that DA treatments did not reduce forskolin-activated [cAMP]_i_. We infer that hemocytes express a functional CsDOP1, but not a CsDOP2 or CsDOP3 receptor.

### Functional characterization of CsDOP1

**Figure 3:**
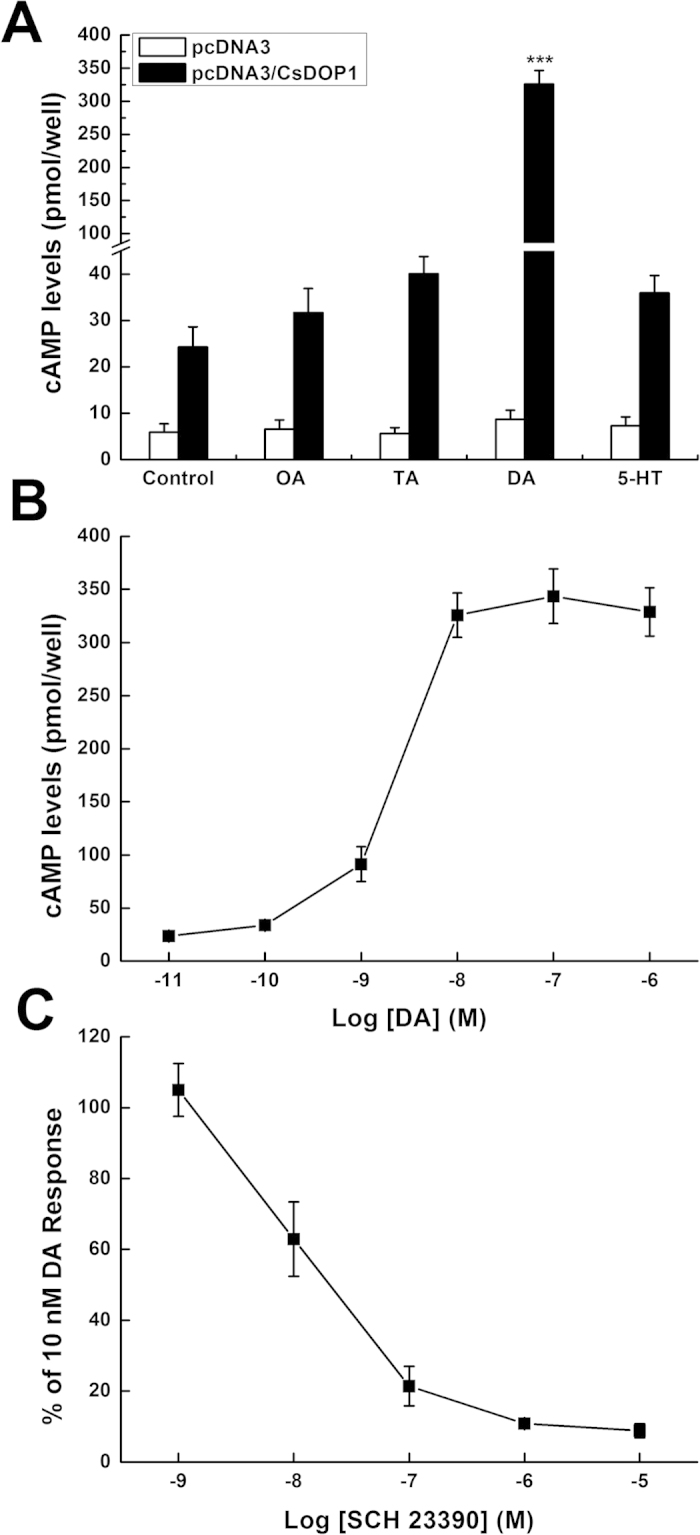
Modulation of intracellular cAMP levels in HEK 293 cells stably expressing the pcDNA3 and pcDNA3-CsDOP1 receptor. Data represent the means ± s.e.m. of at least three independent experiments, each performed in triplicate. The statistical analysis is based on a one-way ANOVA followed by Tukey’s multiple comparison test; ***P ≤ 0.001, **P ≤ 0.01, *P ≤ 0.05. (**A**) Effects of various biogenic amines (10 nM) were examined on intracellular cAMP levels in HEK 293 cells stably transfected with pcDNA3 or pcDNA3-CsDOP1. (**B**) Dose–response relationships of the effects of dopamine on intracellular cAMP levels in HEK 293 cells stably transfected with pcDNA3/CsDOP1. (**C**) Inhibitory effects of SCH23390 on DA-stimulated cAMP levels in HEK 293 cells stably transfected with pcDNA3/CsDOP1.

We stably expressed the CsDOP1 in mammalian HEK 293 cells^27^,^36^,^37^,^38^,^39^ and treated the cells expressing pcDNA3 and, separately, pcDNA3/CsDOP1 with 10 nM octopamine (OA), tyramine (TA), DA and serotonin (5-HT), Our results show that DA, but not the other compounds, significantly increased cAMP production in pcDNA3/CsDOP1-expressing cells, but not in pcDNA3-expressing cells (Fig. 3A). Figure 3B shows that DA treatments led to increased [cAMP]_i_ in a dose-dependent manner with an EC_50_ value of 1.93 × 10^−9^ M. We determined the influence of SCH23390, a specific dopamine receptor type 1 antagonists^37^,^40^, on DA-stimulated cAMP production in CsDOP1-expessing HEK 293 cells (Fig. 3C). SCH23390 inhibited DA-stimulated cAMP production by about 89% (at 10^−6^ M) and by 91% (at 10^−5^ M).

### Exogenous DA leads to rapid phosphorylation of ERK

**Figure 4:**
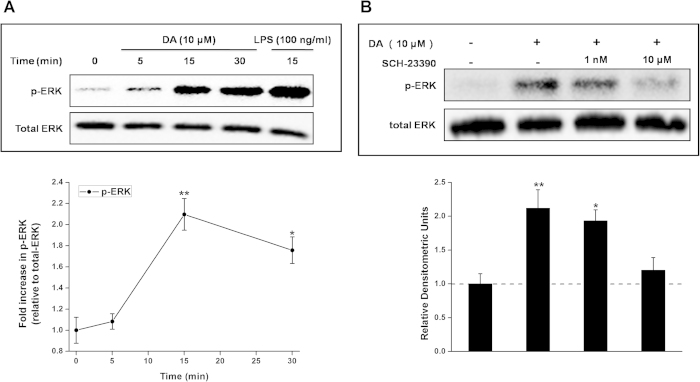
DA induces rapid phosphorylation of ERK1/2 that is inhibited by a DOP1 receptor–selective antagonist. (**A**) Freshly isolated naive hemocytes were incubated with exogenous DA (10 μM) at 25 °C. Samples were lysed, immunoblotted, and probed for phospho-ERK1/2 (top). Blots were stripped and reprobed for total ERK1/2 to confirm equal loading in each lane (bottom). Densitometric analysis was performed showing a maximal increase in phospho-ERK1/2 at 5 minutes following stimulation with exogenous DA (relative to total ERK). (**B**) Freshly isolated hemocytes were incubated with SCH 23390 (DOP1 receptor antagonist) for 1 hour, 25 °C. Samples were then pulsed with DA (10 μM) for 5 minutes at 25 °C and analyzed for phospho-ERK1/2 (top) and total ERK1/2 (bottom). Densitometric analysis was performed, showing phospho-ERK p42 (relative to total ERK). Data represent mean ± S.D. (error bars) of three to five experiments. The statistical analysis is based on a one-way ANOVA followed by Tukey’s multiple comparison test; **P ≤ 0.01, *P ≤ 0.05.

We investigated the time course of ERK phosphorylation in DA-treated hemocytes. ERK phosphorylation increased above basal levels within 5 min and peaked between 10 and 20 min after DA treatment (at 10 μM; Fig. 4A). We confirmed the action of CsDOP1 in ERK activation by pretreating hemocytes with the most potent CsDOP1 receptor antagonist, SCH23390 (at 1 nM and 10 μM). Figure 4B shows that 10 μM, but not 1 nM, SCH23390 fully inhibited DA-induced ERK phosphorylation.

### Inhibition of DA signaling impairs hemocyte phagocytosis

**Figure 5:**
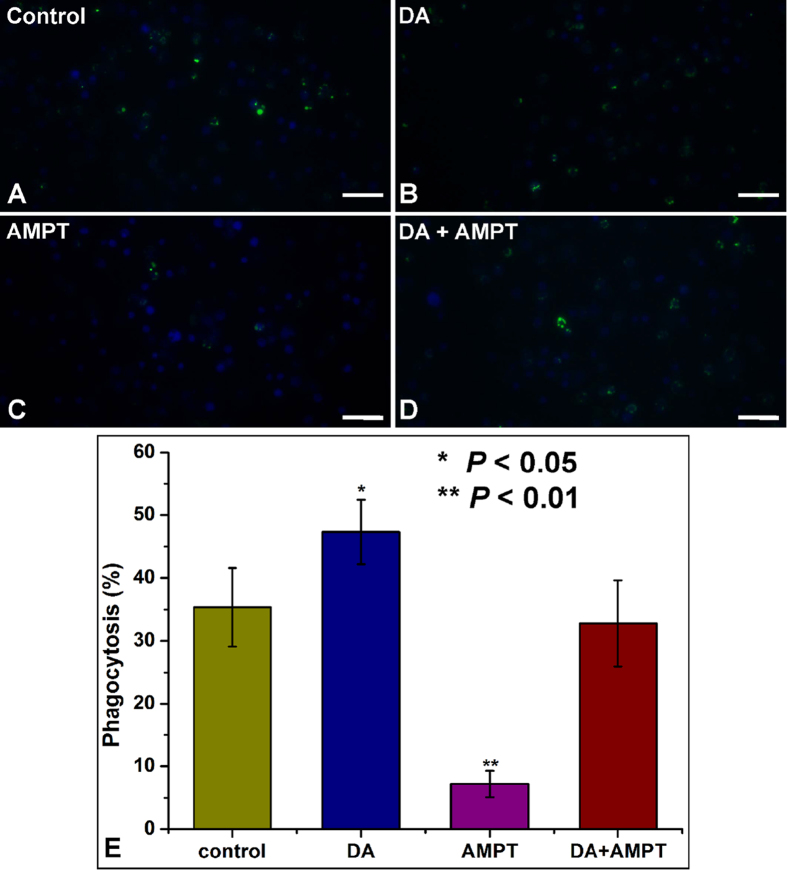
Requirement for DA in phagocytosis of FITC-labeled *E. coli*. (**A**–**D**) Phagocytosis of FITC-labeled *E. coli* by mock- (**A**), DA-treated (**B**), AMPT-treated (**C**) or DA and AMPT-treated (**D**) hemocytes. Scale bars represent 200 μm. (**E**) A graph summarizing the quantification of these assays with untreated-hemocytes as a control. The statistical analysis is based on a one-way ANOVA followed by Tukey’s multiple comparison test; ***P ≤ 0.001, **P ≤ 0.01, *P ≤ 0.05.

We treated hemocyte preparations with α-methyl-DL-tyrosine methyl ester hydrochloride (AMPT), a tyrosine hydroxylase inhibitor^41^ and recorded substantial reductions in the basal DA synthesis compared with controls. We saw in Fig. 1C that LPS treatments led to increased DA concentrations. Figure S2 shows that LPS did not lead to increased DA in AMPT-treated hemocyte preparations. DA enhanced the phagocytosis in untreated, but not in AMPT-treated hemocyte preparations (Fig. 5). The rescue experiment showed that DA plus AMPT did not influence phagocytosis relative to untreated control hemocytes (Fig. 5D,E).

**Figure 6:**
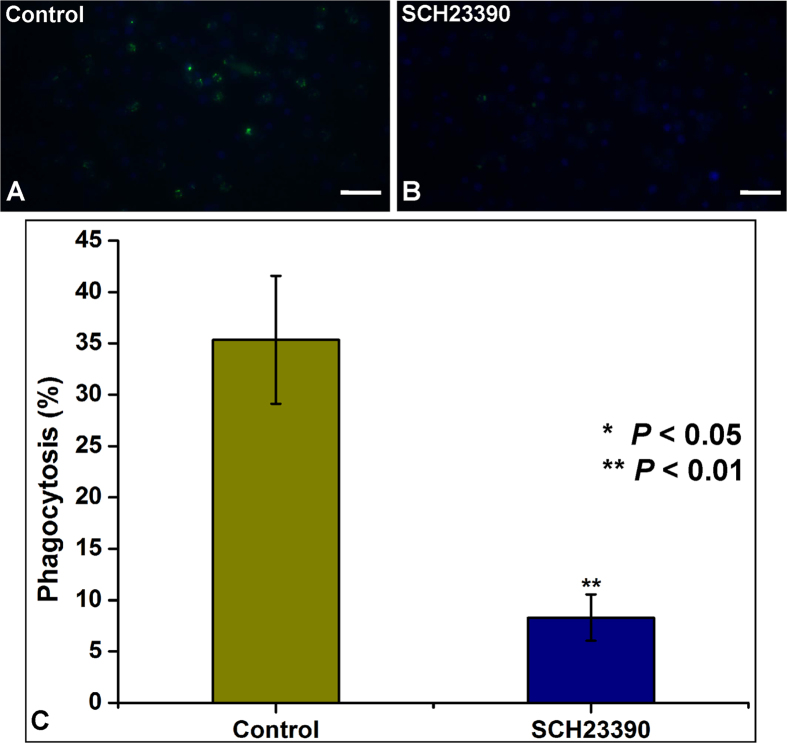
Requirement for DOP1 in phagocytosis of FITC-labeled *E. coli*. (**A**,**B**) Phagocytosis of FITC-labeled *E. coli* by control- (**A**) and SCH23390-treated (**B**) hemocytes. (**C**) A graph summarizing the quantification of these assays with untreated-hemocytes as a control. The statistical analysis is based on a one-way ANOVA followed by Tukey’s multiple comparison test; ***P ≤ 0.001, **P ≤ 0.01, *P ≤ 0.05.

Treating hemocyte preparations with the potent CsDOP1 receptor antagonist, SCH23390, led to impaired phagocytosis (Fig. 6).

### DA rescued mortality following fungal challenge

**Figure 7:**
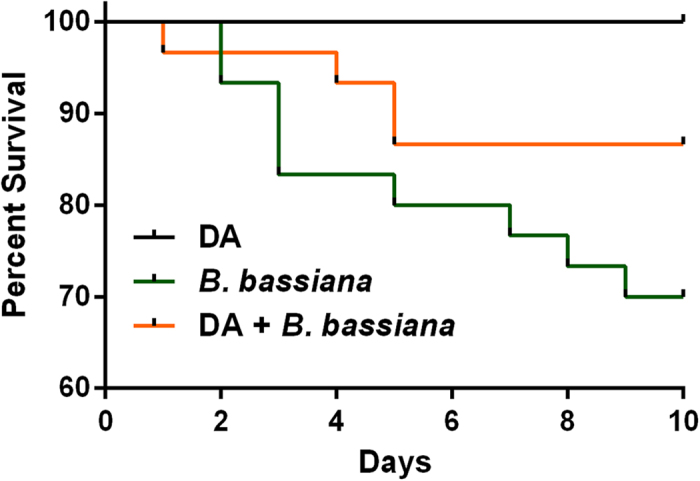
Effect of dopamine on the survival rate of *Chilo suppressalis* challenged with *Beauverua bassiana*. The curves of control treatments displayed as a single black line. (Five replicates for each treatment, *χ*^2^ = 10.87, *P* < 0.01).

Fungal inoculation (*Beauveria bassiana*) was performed by immersing the insect body into a spore solution. DA was applied topically on the middle-abdomenal notum to avoid septic injury. The unchallenged, control RSBs received DA at 1025 ng/larva and were then treated with PBS; all control insects survived (Fig. 7). Survivorship was reduced to about 70% at 9 days post challenge in larvae without DA rescue treatment; fungal challenge with a subsequent DA rescue treatment increased survival to about 88% from day 5 to day 10 (*χ*^2^ = 10.87, *P* < 0.01; Kaplan-Meier analysis)

## Discussion

The results reported in this paper firmly support our hypothesis that DA is one of the signal moieties responsible for mediating phagocytosis by insect hemocytes. Several lines of data form a complete argument. First, challenging RSBs with a fungal pathogen led to substantial larval mortality, which was reduced in larvae treated with DA. Second, DA is present in RSB plasmatocytes and granulocytes, the lepidopteran immune-conferring hemocytes. Third, *in vitro* challenge with bacterial LPS stimulated DA production in experimental hemocytes and led to increased expression of genes encoding TH and DDC, the two enzyme responsible for DA biosynthesis, shown by increased levels of the cognate mRNA transcripts and protein. The hemocytes were immunocompetent because LPS challenge led to expression of genes encoding two immune defense proteins, defensin and PGRP-S2. Fourth, hemocytes constitutively express a gene encoding the DA receptor, CsDOP1, again shown by the presence of the receptor transcripts and protein. Our immunohistochemical protocol shows the receptor is located in the hemocyte plasmamembranes, as expected for GPCRs. Fifth, the DA-activated CsDOP1 couples with a G_s_ protein, which leads to increased [cAMP]_i_s in a dose-dependent manner. We also show that DA treatments did not reduce the high, forskolin-stimulated [cAMP]_i_s and that DA treatments did not influence intracellular Ca^2+^ concentrations. Sixth, we expressed the CsDOP1 receptor in HEK 293 cells and show that DA, but not OA, TA or 5-HT, stimulated increased [cAMP]_i_s. Seventh, DA treatments led to a rapid increase in ERK phosphorylation, which was inhibited by the CsDOP1 receptor antagonist, SCH23390. Eighth, DA stimulated phagocytosis in *in vitro* hemocyte preparations, which was inhibited in preparations treated with the TH inhibitor AMPT. The elements in this summary form a powerful array of evidence supporting our view that DA signals phagocytosis in hemocytes of microbially challenged RSBs.

Our mortality data show that the fungal challenges in untreated larvae led to decreased survivorship, however, a considerable number of larvae, about 70%, survived. We infer that the cellular immune functions operated during the fungal challenges and that cellular immune signaling effectively protected the infected larvae. The DA treatments enhanced survivorship. This result gives additional credence to our view that DA is produced in hemocytes and acts in an autocrine, or perhaps paracrine mechanism to increase cellular defense reactions to infection. Our view of an auto- or paracrine mechanism is supported also by presence of DA in hemocytes. We detected DA in unchallenged hemocytes and registered infection-driven increases in expression of genes encoding TH and DDC and increases in DA synthesis. Hemocytes constitutively express a plasmamembrane DA receptor, CsDOP1, indicating that individual hemocytes are equipped to respond to DA released from themselves or neighboring hemocytes. It was also reported that prostaglandins (PGs) act in auto- and paracrine mechanisms^42^ and they influence insect hemocyte phagocytosis, likely in a similar auto-and paracrine manner^1^,^43^.

**Figure 8:**
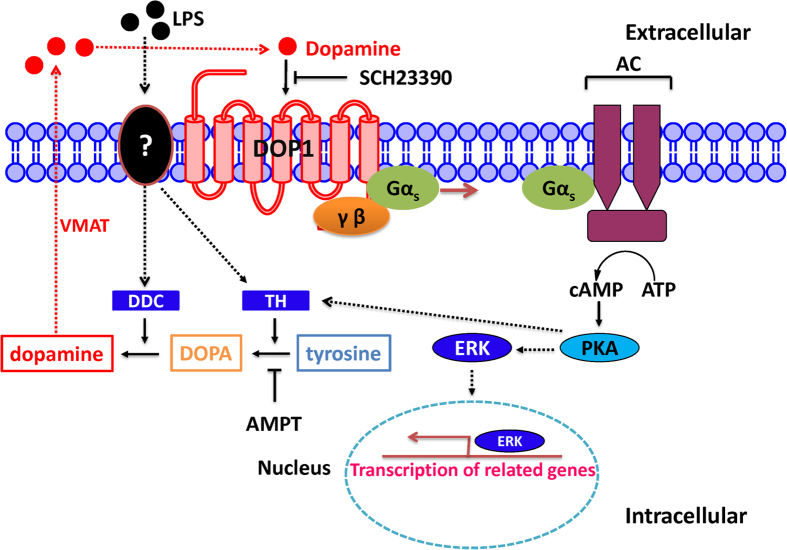
A schematic diagram of the potential pathways involved in dopamine-mediated hemocyte phagocytosis. LPS activated the receptor of hemocyte membrane (unknown receptor) and then enhanced the expression of TH and DDC, which catalyzed tyrosine into dopamine. Dopamine, which secreted from hemocyte, activated the hemocyte-membrane receptor DOP1 and then might initiate the phagocytosis-related immune response. Abbreviation: DAT: dopamine transporter; AC: adenylate cyclase; VMAT: vesicular monoamine transporter.

Our data indicated that when the hemocytes were challenged with LPS. The unknown receptor which located on the membrane of hemocyte will be activated. It might enhance the expression of dopamine-producing enzymes (TH and DDC). The generated DA might be transported from intracellular into extracellular by vesicular monoamine transporter (VMAT). Finally, the DA signaling pathway is immediately launched . DA binds with its hemocyte receptor, CsDOP1 and then the intracellular component couples with a Gs protein that activates adenylyl cyclase to increase [cAMP]_i_s was also initiated. We find that hemocyte-expressed CsDOP1 receptor are functional. Signaling through the CsDOP1 receptor might induces the seconder messenger phospho-ERK and activates the transcription of immune-related genes (Fig. 8). It might be thought that other DA receptors act in phagocytosis, however, we deem that an unlikely possibility because we found that DA did not reduce forskolin-driven high cAMP concentrations in *in vitro* hemocyte preparations, nor did DA treatments lead to release of Ca^2+^ from intracellular stores. We infer a single DA receptor, CsDOP1, but not other DA receptors, acts in hemocyte phagocytosis. Our pharmacological data similarly indicates a single DA receptor type. While DA stimulated ERK phosphorylation, we found the DA receptor antagonist, SCH23390 cancelled the DA effect. SCH23390 is a highly selective vertebrate D1 receptor antagonist^44^. The selectivity of SCH23390 and high sensitivity for one agonist over others indicates a single DA receptor type in RSB hemocytes and likely most lepidopteran hemocytes.

The idea that DA acts in insect immunity has been recognized. Sideri *et al.* (2008) reported on the presence of DDC associated with medfly, *Ceratitis capitata* hemocytes^45^. They found that siRNA silencing a gene encoding DDC blocked phagocytosis and suggested that phagocytosis and nodulation depend on DA and DA-derived compounds. Similarly, in their work on the *Cotesia kariyai/Pseudaletia separata* host/parasitoid relationship, Noguchi *et al.* (2003) found increased concentrations of DA are associated with increases in the insect cytokine, growth-blocking peptide (GBP)^46^. They found that a gene encoding DDC (converts dopa to dopamine, Fig. 1) is expressed at low levels in epidermis, brain and hemocytes; GBP injections led to increased expression of DDC in all three tissues. Although they did not report on the influence of DA on hemocyte behavior, the authors indicate that DA acts in host/parasitoid relationships. Similarly, Gorman *et al.* (2007) reported that TH activity was up-regulated in tobacco hornworm *Manduca sexta* hemocytes and fat body following immune challenge^35^. This is in concert with our supplementary data showing that treating RSB hemocyte preparations with the TH inhibitor, AMPT, effectively blocked DA production. In their model, DA produced after infection is incorporated into melanin. The melanin can be used in melanization of nodules and other defense actions. We infer DA has at least two general roles in insect immune reactions, one as a signal moiety in hemocytic phagocytosis and another as a structural component of melanin.

DA-mediated phagocytosis is likely a general insect cellular defense mechanism because DA treatments mitigated the lethality of a fungal pathogen *B. bassiana* and challenging hemocytes with LPS prepared from the bacterium, *E. coli,* led to increased expression of genes encoding TH and DDC, increased DA production and increased phagocytosis. Several other signal moieties also mediate insect cellular immune functions. The biogenic amines, octopamine and serotonin, signal phagocytosis and nodulation in cockroach, *Periplaneta americana* hemocytes^47^; the insect cytokine, plasmatocyte spreading peptide (PSP) stimulates hemocyte spreading on surfaces. Several groups have reported that prostaglandins (PGs) and other eicosanoids mediate a number of hemocyte actions, including phagocytosis, nodulation, cell spreading and cell migration^48^. These findings prompt the issue of how are these several mediators integrated to produce the observable hemocytic defense behaviors. Kim and his colleagues^48^,^49^ proposed a model in which cytokines, PGs, biogenic amines and possibly other signal molecules act via specific GPCRs, all of which stimulate the small G-protein, Rac1. Rac1 activates the first step in biosynthesis of prostaglandins and other eicosanoids, phospholipase A_2_ (PLA2), leading to PG biosynthesis and hemocytic responses. We speculate the CsDOP1 similarly acts via Rac1 in RSB hemocytes.

## Methods

### Organisms and drugs

The larvae of the rice stem borer, *Chilo suppressalis* were collected from fields in Fuyang, Zhejiang Province, China. They were reared using rice seedling method reported by Shang *et al.*^50^ under the conditions of 27 ± 1 °C, relative humidity (R.H.) of 70–80% and a photoperiod of 16:8 (light: dark) h, except for adult mating and oviposition at 85–90% R.H. The insect pathogenic fungus, *Beauveria bassiana* was obtained from Prof. Jianya Su’s lab and maintained by PDA medium. All drugs were purchased from Sigma-Aldrich (St Louis, MO, USA) unless stated otherwise.

### Hemocyte isolation and stimulation

The fifth-instar larvae of *C. suppressalis* were surface sterilized with 70% ethanol. The proleg was cut with a pair of scissors and the hemolymph was collected in Grace’s medium (1:10, v/v; Invitrogen, Carlsbad, CA). Hemocytes were treated with 50 or 100 ng of lipopolysaccharides (LPS) (*Escherichia coli* 0111:B4; Sigma Aldrich). Supernatants were obtained and stored in liquid nitrogen.

### DA detection

The hemolymph from five fifth-instar larvae was collected and mixed. The combined hemolymph (30 μL) was mixed with 170 μL Grace’s Insect Medium (as described above) containing 50 μg/mL tetracycline and 2 μL saturated 2-phenylthiourea (PTU). The diluted hemolymph was added to each well of an 8-well chambered coverglasses (Lab-Tek™, Nunc, Thermo Fisher Scientific, Rochester, USA) and hemocytes were allowed to adhere to the slide for 20 min at 25 °C to form monolayers. Then, hemocytes were fixed with 4% paraformaldehyde. Dopamine (DA) was labeled with anti-dopamine antibodies (ab6427; Abcam, Cambridge, U.K.) for 72 hours at room temperature, followed by biotinylated anti–rabbit Ig for 24 hours at 4 °C and SA-Alexa Fluor 546 (Invitrogen) for 1 hour at room temperature. The nuclei of hemocytes were stained with 1 μg/mL of 4′-6-diamidino-2-phenylindole (DAPI, Beyotime Biotech, Jiangsu, China) for 5 min and hemocytes were observed by fluorescent microscope (Zeiss, Göttingen, Germany).

Dopamine in the supernatants of hemocytes was quantified with reverse-phase high performance liquid chromatography (HPLC) and mass spectrometry (MS) analysis. Briefly, the supernatants were filtered by 0.45 μm filter, transferred to 1.5 ml Eppendorf tubes and stored at −80 °C until MS analysis. 20 μl supernatant was automatically loaded into Inlet Systems (Agilent Technologies1200 Series RRLC) with a C18 reversed phase column (Eclipse Plus C18, 3.0 × 100 mm, 1.8 μm). An Agilent 6460 mass selective detector with an Agilent Jet Stream (AJS) (G1958-65138) ion source (Agilent Technologies, Palo Alto, CA, USA) was used. Instrumental settings for the Agilent6460 mass selective detector included: gas temperature: 350 °C, gas flow: 4 l/min, sheath gas temperature: 350, sheath gas flow: 12 l/min, nebulizer pressure: 344.8 kPa, capillary voltage: 3500 V, nozzle voltage: 0 V in negative mode and 500 V in positive mode. Nitrogen gas was used as the drying and collision gas for all LC–MS/MS instruments. Data analysis was performed using Agilent Mass Hunter B.02.01 software. Dopamine was quantified by reference to external standards.

### RNA isolation and qPCR

Total RNA was extracted from hemocytes with Trizol reagent (Life Technologies) in accordance with the manufacturer’s instructions. Reverse transcription was performed with 1 μg of RNA by using a ReverTra Ace^®^ qPCR RT kit (Toyobo, Osaka, Japan). Real-time quantitative PCR was then performed on cDNA preparations using the SsoFast Eva Green Supermix with Low Rox (Bio-Rad, Hercules, CA) and Applied Biosystems 7500 Real-Time PCR System (Applied Biosystems by Life Technologies, Carlsbad, CA) following the manufacturer’s instructions. The quantification of transcript levels of different genes was conducted according to the 2^−ΔΔCT^ method^51^. The primers used in this study are shown in Table S1.

### Western blot

After isolation and exposure to LPS, hemocytes were lysed in Laemmli buffer (1% Triton X-100, 150 mM NaCl, 10 mM Tris-HCl pH 7.5, 5 mM EDTA, 1 mM sodiumo-vanadate) containing 5% mercaptoethanol. Debris were sedimented by centrifugation (15,500 g, 10 minutes, 4 °C), supernatants were collected, and their protein content was determined (Bio-Rad Laboratories). Samples were then separated in a denaturing polyacrylamide gel and transferred to a nitrocellulose membrane. After blocking (5% Tris-buffered saline pH 7.0 containing 0.1% Tween 20) and washing, membranes were then incubated overnight with primary antibodies against TH, DDC, DOP1 and COMT (1:1,000). Membranes were then incubated with secondary antibody horseradish peroxidase–conjugated goat anti–rabbit IgG diluted 1:5,000 in TBS-T. Membranes were rinsed 3 times with wash buffer and then incubated with with the ECL SuperSignal System (Pierce, USA). Membranes were stripped and reprobed with antibodies against Actin (Abcam) for 3 hours at room temperature, followed by incubation with secondary antibody. Immunoblot signals were quantified using a Bio-Rad GS-700 Image densitometer and analyzed using Molecular Analyst (v2.1.2; Bio-Rad). Densitometry units for each band are expressed as OD adjusted by surface (OD × mm^2^).

### Immunofluorescence

For immunofluorescence of CsDOP1, it is the same with DA detection except using the anti-CsDOP1 as the primary antibody.

### Intracellular Ca^2+^ and cAMP assays

Ca^2+^ imaging and cAMP assays were conducted as previously described^52^.

### ERK phosphorylation

Hemocytes (2.5 × 10^4^ cells/μL) were stimulated with DA (10 μM) for 5, 15, or 30 minutes at 25 °C. In some experiments DOP1 receptor signaling was blocked by preincubation with the antagonist, SCH 23390 (Sigma; S7389) at the concentration of 1 nM and 10 μM for 60 minutes at 25 °C. Cell lysates were prepared, and proteins were resolved by SDS-PAGE. Blots were immunolabeled overnight at 4 °C using 1 μg/mL polyclonal rabbit Phospho-p44/42 MAPK (Erk1/2) (Thr202/Tyr204) Antibody (Cell Signaling Technology, Beverly, MA) diluted in TBS-T. Secondary labeling, detection, and analysis were performed as previously described. Membranes were stripped and reprobed with polyclonal rabbit p44/42 MAPK (ERK1/2) antibody (1 μg/mL; Cell Signaling Technology).

### Phagocytosis assay

The assay for phagocytosis was performed according to the method described by Huang *et al.*^52^ and Wu *et al.*^53^ with minor modifications. Briefly, larval hemocytes (2 × 10^5^ cells) were incubated in 45 μl Grace’s medium containing FITC-labeled *E. coli* (Invitrogen) with 10 bacteria per hemocyte, in the presence of drugs solution or control solution (5 μl) for 30 min at 26 °C in a 96 well tissue culture plate (Nunc, Roskilde, Denmark). After incubation in darkness in a moist chamber, internalized FITC-labeled *E. coli* was measured by quenching attached FITC-labeled *E. coli* with 200 μl of PBS containing Trypan blue (0.4%). After washing each well three times with PBS, hemocytes were treated with 4% formaldehyde for 15 min to fix the monolayer. The nuclei of hemocytes were also stained with 1 μg/mL of DAPI (Beyotime Biotech). The proportion of cells that had phagocytosed labeled *E. coli* was determined under a Florescence microscope (TS100, Nikon, Tokyo, Japan) in fluorescence mode at 200 × magnification in five different fields.

### Survival analysis

To dilute DA, we used acetone and PBS buffer (pH 7.2) with 1:1 (v/v) mixture as solvent. Fourth-instar larvae of rice stem borer (8–9 mg) were topically treated with 1 μl of DA solution (1025 ng/μl) onto the middle-abdomen notum of the larvae with a hand micro-applicator (Institute of Plant Physiology and Ecology, Chinese Academy of Sciences, Shanghai, China). After twenty minutes, the rice stem borers were immersed in a spore suspension (1 × 10^6^ spores/ml) of the *Beauveria bassiana* for ten seconds. Rice stem borer that received DA at 1025 ng/larva and then PBS served as the unchallenged controls. There were three treatments and each treatments was conducted with 30 larvae. The experiment lasted 10 days.

### Statistical analysis

All values are expressed as means ± standard error (s.e.m.). Data were analyzed using the analysis of variance (ANOVA) with Tukey–Kramer post-hoc test (*P < 0.05, **P < 0.01, ***P < 0.001). Before analysis, the percentage data were normalized using an arcsine transformation. All curve fitting and statistical calculations were performed with Origin 8.0 (Origin Lab, Northampton, MA, USA). EC_50_ is the agonist concentration that evoked the half-maximal response.

## Additional Information

**How to cite this article**: Wu, S.-F. *et al.* Dopamine modulates hemocyte phagocytosis via a D1-like receptor in the rice stem borer, *Chilo suppressalis.* *Sci. Rep.* **5**, 12247; doi: 10.1038/srep12247 (2015).

## Supplementary Material

Supplementary Information
