# Supplementary material for: Dopamine modulates hemocyte phagocytosis via a D1-like receptor in the rice stem borer, Chilo suppressalis
Source: Sci Rep. 2015 Jul 16;5:12247. doi: 10.1038/srep12247 (PMC4503959; doi:10.1038/srep12247)
Supplement: Supplementary Information [file srep12247-s1.pdf]

Supplementary information:

Dopamine modulates hemocyte phagocytosis via a D1-like receptor in the rice stem borer, *Chilo suppressalis*

By

Shun-Fan Wu[1,2], Gang Xu[1], David Stanley[3], Jia Huang[1]*, Gong-Yin Ye[1]*

Authors' name and institution:

[1]State Key Laboratory of Rice Biology & Key Laboratory of Agricultural Entomology of Ministry of Agriculture, Institute of Insect Sciences, Zhejiang University, Hangzhou 310058, China

[2]College of Plant Protection, Nanjing Agricultural University, Nanjing 210095, China; State & Local Joint Engineering Research Center of Green Pesticide Invention and Application

[3]USDA – Agricultural Research Service, BCIRL, 1503 S. Providence Road, Columbia MO 65203 USA

* Corresponding author. Tel.: +86 571 8898 2696; fax: +86 571 8898 2988.

Address correspondence to: Dr. Gong-Yin Ye and Jia Huang, Institute of Insect Sciences, Zhejiang University, Yuhangtang Road 688, Hangzhou 310058, China

Correspondence and requests for materials should be addressed to: G.Y.Y. (chu@zju.edu.cn) () or J.H. (huangj@zju.edu.cn)

Supplementary Tables S1-S2 and Figures S1-S3

Table S1. The primers used in this study.

Primers	Primer sequences	Accession numbers
For real-time PCR		
CsTH-qRTF	TGTCTTCGATTGGTGAGCTG	KP657623
CsTH-qRTR	TCGACTGTGTCCAGCACTTC	
CsDDC-qRTF	AAGAACAGCACCCTGATTGG	KP657625
CsDDC-qRTR	AAGGGTATGAGCCCGTTCTT	
CsDefensin-qRTF	GCGCGTAATACCGTTTGTCT	GAJS01018928
CsDefensin-qRTR	CGCAAAGGCCATAGGAATAG	
CsPGRP-S2-qRTF	AGTTCCACCAGAATGGCAAC	GAJS01023399
CsPGRP-S2-qRTR	TTTCGATCAAAGCCCTAACG	
CsDOP1-qRTF	GCCAGACAACAGGACCAA	KP784317
CsDOP1-qRTR	GGGACCCAGCACAGTAAG	
CsDOP2-qRTF	ACTAAGCAAGTGATGAGACC	KP784318
CsDOP2-qRTR	CGACAGTCCGTTATTTTGC	
CsDOP3-qRTF	GCTTTCGTGTGCGATGTCTA	KP784319
CsDOP3-qRTR	CAACCACACCAGCACTATGG	
Elongation factor-1 (EF-1)-F	TGAACCCCCATACAGCGAATCC	Hui et al. 2011 [1]
Elongation factor-1(EF-1)-R	TCTCCGTGCCAACCAGAAATAGG	
For eukaryotic expression		
CsDOP1- HindIII-F	CCC***AAGCTT***ACCATGGAGTTCG	
CsDOP1-Xho1-R	CCG***CTCGAG***TCAGATGTGATAC	

Table. S2 Ct values of CsDOP2 and CsDOP3 using hemocyte of rice stem borer as qPCR template.

Receptors	Ct values								Mean Ct values	S.E. of Mean
CsDOP2	34.85	34.01	35.09	35.24	36.77	34.79	34.33	33.61	34.80	0.30
CsDOP3	33.94	34.92	33.90	34.02	33.51	35.51	33.75	34.21	34.21	0.21

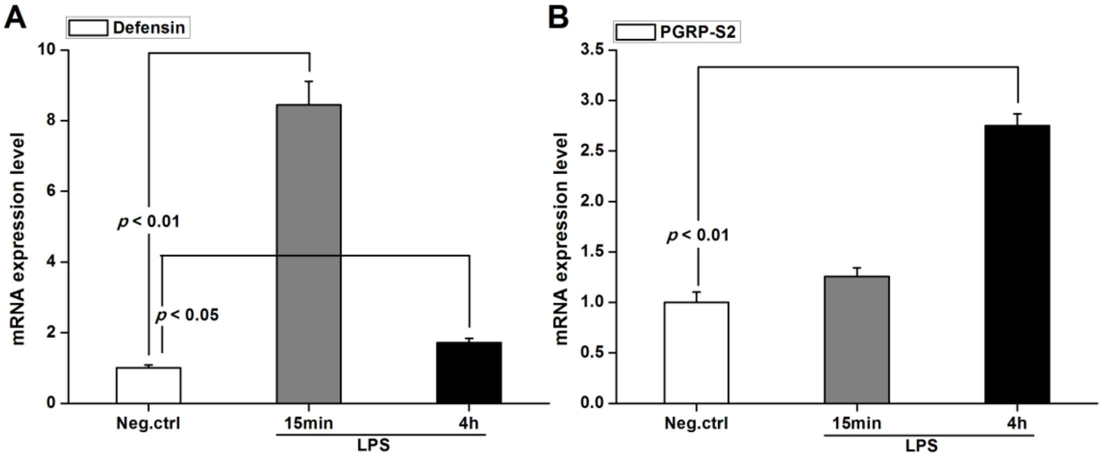

Figure S1. Defensin and PGRP-S2 was used as positive control. After isolation and stimulation of hemocytes with 100 ng/ml of LPS *in vitro*, mRNA from hemocytes was isolated and subjected to real-time PCR analysis for defensin (A) and PGRP-S2 (B). Real-time PCR data are presented as means ± s.e.m.; $n \geq 6$ per bar. Neg. ctrl: negative control.

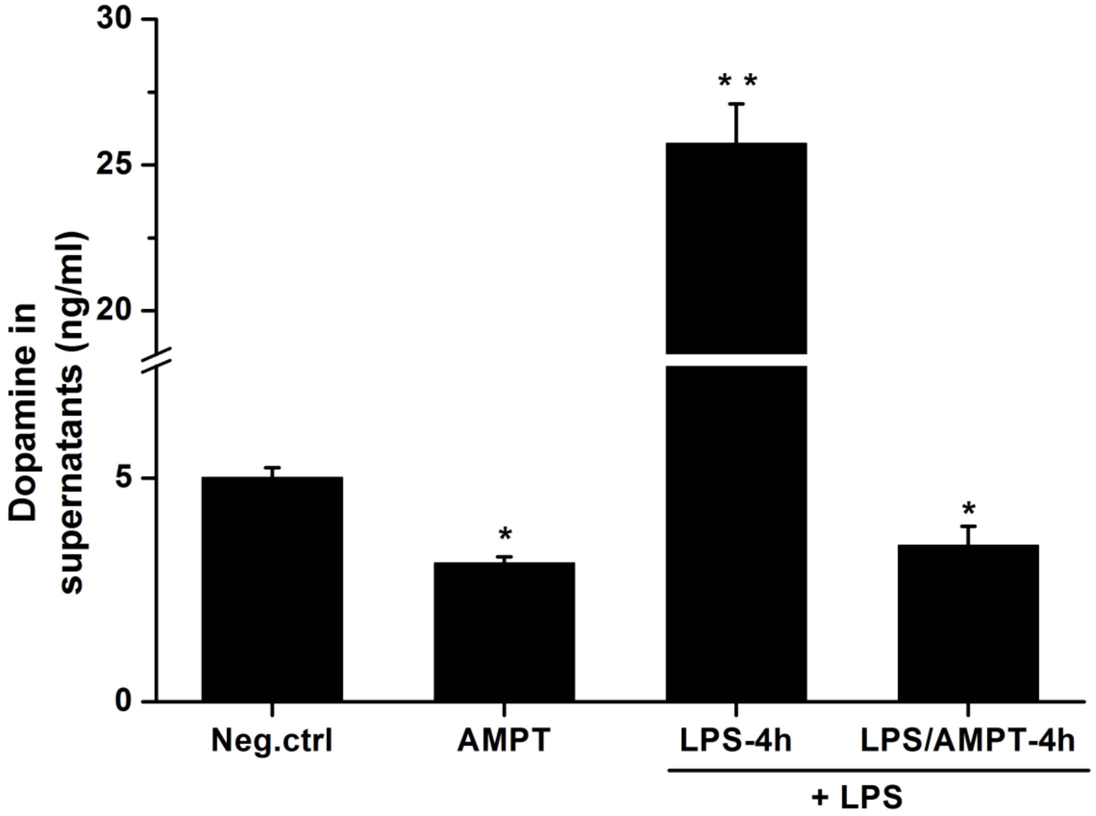

Figure S2. The amount of dopamine in the supernatant of the hemocytes. Asterisk shows statistically significant differences between means (mean + s.e.m); $^{**}P \leq 0.01$, $^{*}P \leq 0.05$.

1. Hui, X.M. et al. RNA interference of ace1 and ace2 in *Chilo suppressalis* reveals their different contributions to motor ability and larval growth. *Insect Mol. Biol.* **20**, 507-518 (2011).
